# Spatial Intensity Distribution Analysis: Studies of G Protein-Coupled Receptor Oligomerisation

**DOI:** 10.1016/j.tips.2017.09.001

**Published:** 2018-02

**Authors:** John D. Pediani, Richard J. Ward, Sara Marsango, Graeme Milligan

**Affiliations:** 1 Centre for Translational Pharmacology, Institute of Molecular, Cell and Systems Biology, College of Medical, Veterinary and Life Sciences, University of Glasgow, Glasgow G12 8QQ, UK

**Keywords:** G protein-coupled receptor, dimerisation, SpIDA, quaternary structure, ligand regulation

## Abstract

Spatial intensity distribution analysis (SpIDA) is a recently developed approach for determining quaternary structure information on fluorophore-labelled proteins of interest *in situ*. It can be applied to live or fixed cells and native tissue. Using confocal images, SpIDA generates fluorescence intensity histograms that are analysed by super-Poissonian distribution functions to obtain density and quantal brightness values of the fluorophore-labelled protein of interest. This allows both expression level and oligomerisation state of the protein to be determined. We describe the application of SpIDA to investigate the oligomeric state of G protein-coupled receptors (GPCRs) at steady state and following cellular challenge, and consider how SpIDA may be used to explore GPCR quaternary organisation in pathophysiology and to stratify medicines.

## Trends

GPCRs may exist and function as monomers: however, abundant evidence suggests they can form dimers/oligomers.

This concept has implications for drug discovery as it may offer opportunities to modulate the effects of known pharmaceuticals or identify new drug therapies.

A variety of approaches have been applied to this issue from traditional biochemical techniques, via resonance energy transfer approaches to recently developed image analysis-based techniques such as SpIDA. This uses mathematical analysis of confocal microscopy images to generate quantal brightness and density information for a fluorophore-tagged receptor.

SpIDA can be applied to live or fixed cells and native tissue.

SpIDA has been applied to GPCRs from each of the major subfamilies to explore their oligomerisation status at steady state and their regulation by receptor density and ligand binding.

## SpIDA: An Approach to Detect Protein Oligomerisation State

**Figure 1:**
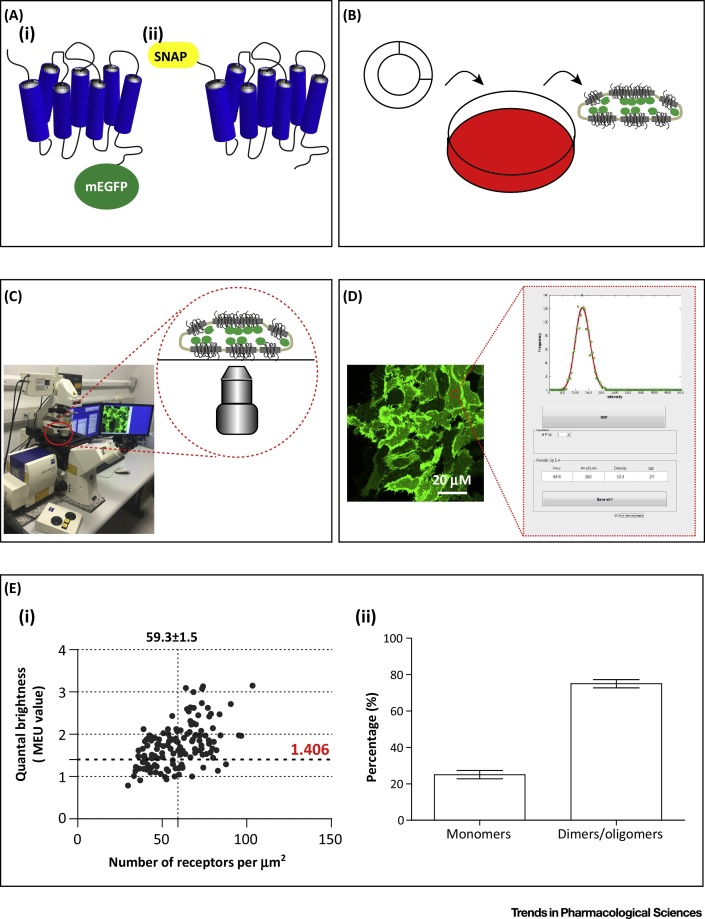
SpIDA Procedure. Schematic representation of the procedure to perform SpIDA measurements as described in Box 1 and in more detail in Ward *et al.*[9]. (A) The G protein-coupled receptor of interest is modified by the incorporation of a fluorophore, for example, monomeric (A^206^K) enhanced GFP (i) or the SNAP-tag (ii), for which labelled substrates are available 43, 44, at the carboxyl (intracellular) or amino (extracellular) terminus of the receptor, respectively. (B) The receptor construct is expressed stably in a heterologous mammalian cell system such as Flp-In T-REx 293 or CHO-K1 cells. (C) Laser scanner confocal images are collected from the glass coverslip attached to the basolateral membrane of the cells. (D) RoIs (red square) are selected and analysed using MATLAB graphical user interface programme. (E) Mean fluorescence intensity and QB values are normalised to the QB of the fluorophore label in its monomeric state and provides information on the density (expressed as particles per μm^2^) and the oligomeric state (expressed as MEUs) of the fluorophore-tagged protein. (i) The expression levels and calculated oligomeric state of human dopamine D_3_ receptor linked to mEGFP (hD_3_–mEGFP) expressed in Flp-In T-REx 293 cells is shown as an example [7]. The vertical broken line corresponds to the mean receptor number per μm^2^ and the horizontal dotted line corresponds to 1.406 MEU which represents mean +2 standard deviations of the data set (see Marsango *et al.*[7] for details). (ii) RoIs characterised by QB MEU values >1.406 were considered to contain a prevalence of hD_3_–mEGFP in a dimeric/oligomeric state. Data are adapted from Marsango *et al.*[7]. Abbreviations: mEGFP, monomeric enhanced GFP; MEU, monomeric equivalent unit; QB, quantal brightness; RoI, region of interest; SpIDA, spatial intensity distribution analysis.

**Spatial intensity distribution analysis (SpIDA)** (see Glossary) is a recently developed biophysical approach to interrogate the organisational structure of proteins of interest *in situ* 1, 2, 3, 4. SpIDA is based on the generation of pixel-integrated fluorescence intensity histograms, created from regions of interest (RoIs) drawn on recorded confocal images, which are analysed with **super-Poissonian distribution functions** to obtain density maps and **quantal brightness (QB)** values of the fluorophore used to label the protein being studied. Normalisation of these values to the QB of the fluorophore label in its monomeric state provides information on both the density (usually expressed as particles per μm^2^) and the oligomeric state (expressed as monomeric equivalent units, MEUs) of the assessed protein. Here, the advantages and the limitations of SpIDA when compared with other biophysical approaches are briefly considered. This is followed by a description of the use of SpIDA to investigate the oligomeric state of rhodopsin-like (class A) 5, 6, 7, secretin-family (class B) [8], and glutamate-like (class C) [1] **G protein-coupled receptors (GPCRs)** and how this may be modulated by receptor expression level and altered by treatment of cells expressing these receptors with **antagonist** and **inverse agonist** ligands. Box 1 and Figure 1 provide brief overviews of key steps necessary to establish and analyse SpIDA measurements. A fuller description of technical aspects of SpIDA is available in Ward *et al.* [9].

**Figure I:**
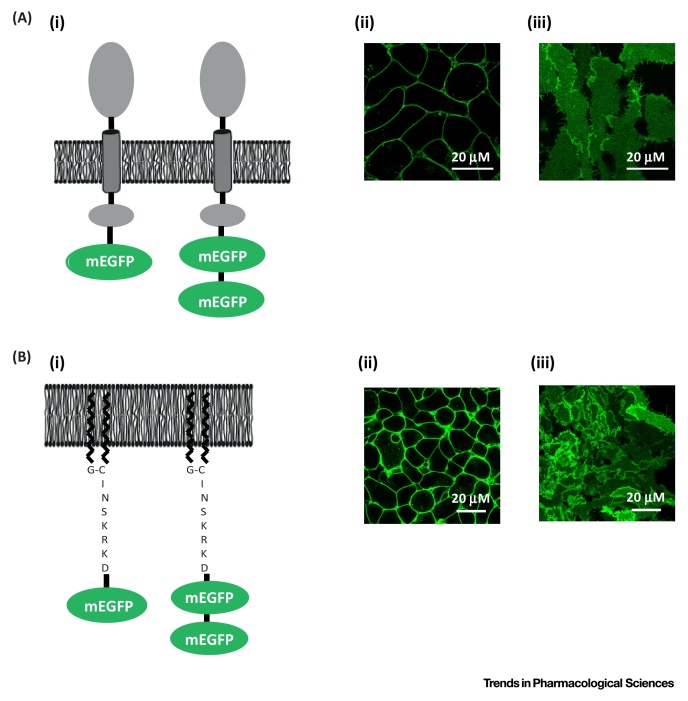
Monomeric EGFP Expression and Localisation to the Plasma Membrane. Schematic representation of a single molecule of mEGFP or a tandem construct of two molecules of mEGFP linked by a short polypeptide. Each was fused to the carboxyl-terminal tail of CD86 (A, i), or to a palmitoylation + myristoylation sequence, derived from the Lyn non-receptor tyrosine kinase, (B, i) and expressed. Laser scanning confocal images of cells expressing a single mEGFP linked to CD86 (A, ii section and A, iii basolateral membrane) or the palmitoylation + myristoylation sequence (B, ii section and B, iii basolateral membrane). Representative images are shown. Abbreviation: mEGFP, monomeric enhanced GFP.

Box 1 **Important Steps to Perform SpIDA Measurements**

Before starting to collect **laser scanning confocal microscope images**, it is necessary to determine certain values specific to the microscope setup in use, such as the laser spot beam waist radius size, to determine the confocal spot resolution, and detector shot noise, to ensure that only the fluorescent intensity fluctuations originating from the excited fluorophore tag are analysed. Recommended image collection parameters for performing SpIDA are detailed in Ward *et al.* [9].

Another critical step is to determine the QB of the fluorescent label that is used to tag the receptor of interest. This requires expression in both a cellular system in which the label is expressed in defined monomeric/dimeric forms and, importantly, in the same cellular compartment as the protein of interest; for example, for GPCR studies, at the plasma membrane. In work described by the authors 5, 6, 7, 8, 40 this has been achieved in two distinct ways (Figure I). In the first a single copy of monomeric EGFP, or a tandem of monomeric EGFP, was fused to the intracellular carboxyl terminus of the single TM, integral membrane protein, CD86. Many studies, using a variety of techniques, have shown CD86 to be strictly monomeric. When expressed, CD86–EGFP is targeted to the plasma membrane. Alternatively, both single and tandem forms of monomeric EGFP were modified at the N terminus by addition of a consensus sequence, derived from the Lyn non-receptor tyrosine kinase N terminus, that resulted in the co- and post-translational addition of the fatty acids palmitate and myristate. Such dual modification is known to anchor otherwise soluble proteins to the plasma membrane. As the C-terminal region of GPCRs is intracellular, then a copy of monomeric EGFP linked in-frame is anticipated to be in an environment akin to that within the control constructs. Initial studies demonstrated that the tandem forms of monomeric EGFP had QB almost exactly twice the value of the single monomeric EGFP linked forms.

Once QB has been calculated for the monomeric/dimeric controls these values are used to normalise experimentally measured QB values for the protein of interest and to generate MEU values that provide information on the oligomeric state of the receptor of interest. In addition, QB values are used to quantify the density of the fluorescent label, often presented as receptors per μm^2^ [9]. Clearly, it is important that the fluorescent label chosen to tag the GPCR of interest does not have inherent tendency to form dimer/oligomers. The web based osFP server (http://codes.bio/osfp/) [45] offers information of this topic and tendency to oligomerise may also be determined as described by Cranfill *et al.* [46].

Appropriate software to perform SpIDA may be downloaded, in the form of a MATLAB graphical user interface programme, from https://neurophotonics.ca/software. Detailed instructions explaining how to operate the SpIDA software can be obtained by downloading the user guide manual from the neurophotonics website. Briefly, this software runs the SpIDA analysis on the RoIs drawn on the input image and generates an Excel compatible text file containing the various analysed numerical parameter output values, along with a reference output image displaying the RoI analysed and fitted curve through the histogram plot.

Alt-text: Box 1

## Advantages and Limitations of SpIDA for Quantification of Protein Oligomeric State

SpIDA was developed in response to limitations of other techniques, including fluorescence correlation spectroscopy (FCS) and photon counting histogram (PCH), used to investigate protein quaternary structure. These techniques base analysis upon temporal fluorescence fluctuations and require that the density and oligomerisation state of the protein in question remains constant during image acquisition [3]. By contrast, SpIDA is based upon a spatial domain fluctuation, recorded as single images representing moments in time of receptor density and oligomerisation state. A significant advantage of SpIDA is that there is less susceptibility to potential effects of photobleaching because only a single input image is required, and hence, underestimation of QB and thus the size of protein complexes as such effects are minimised [3].

An important technical challenge when using fluorescent labels in the determination of protein oligomerisation state is that of subquantitative and therefore, imperfect, labelling (which also applies equally to other approaches). This may be due, for example, to misfolding of a fluorescent protein or inefficient covalent incorporation of a fluorophore or antibody-mediated addition of a fluorescent species. Clearly, this will bias the distribution of oligomeric states measured within a cellular system. To alleviate such concerns Godin and co-workers [10] developed the SpIDA software to include a probability-weighted correction algorithm for nonemitting labels that can be applied when performing SpIDA to correct for nonquantitative labelling.

A limitation of SpIDA is its inability to directly quantify rapid, real-time temporal dynamic diffusion information relating to oligomeric complex formation or dissociation within cellular compartments, including the plasma membrane. Moreover, dynamic protein oligomeric complex formation occurs on a spatial resolution distance scale <100 nm and this separation distance is too small to be resolved by conventional diffraction-limited fluorescence microscopy. To circumvent this limitation, the laser spot confocal volume is oversampled and the excitation illumination volume for membrane oligomerisation measurements is quantified as a surface as opposed to a 3D volume 5, 6, 7, 8, 9.

## Use of SpIDA to Define the Quaternary Structure of GPCRs

That single-polypeptide GPCRs can form and function as dimers and/or higher-order oligomers, rather than simply as monomers, has been hypothesised and then tested over a period of many years [11]. For example, it is clear that formation of either homo- or heterocomplexes defines the pharmacology and function of members of the family of glutamate-like or class C GPCRs [12]. Furthermore, a considerable number of studies have been directed at defining the quaternary structure of class B GPCRs, with particular focus on the **secretin receptor**. Mutagenesis studies and the use of peptides corresponding to each of the transmembrane domains (TMs) of the secretin receptor resulted in the conclusion that it exists as a dimer stabilised by interactions that occur between residues in TM IV 13, 14 of each protomer. Interestingly, disruption of the dimeric quaternary structure of the secretin receptor was found to correlate with reduced potency of secretin to stimulate intracellular cAMP production [13], and the idea that disruption of signalling effectiveness by altering the quaternary structure of other GPCRs is a driver for better understanding of the basic rules that define such interactions.

Unlike class C GPCRs, even the existence, and certainly the relevance, of dimers and/or higher-order oligomers of members of the rhodopsin-like or class A family of GPCRs is still subject to debate. Although class A receptors can be purified as monomers and, when reconstituted as monomers, be shown to be functional 15, 16, there is increasing evidence to indicate that these receptors can interact to form dimers and higher-order oligomers and that this property can influence various aspects of receptor signalling such as trafficking, ligand binding, G protein coupling, and internalisation from the cell surface 11, 17, 18, 19, 20, 21. Despite this expanding body of knowledge, there are key questions about the mechanism and roles of class A GPCR dimerisation that remain unresolved. Examples include the extent of such interactions, whether this is controlled by receptor expression levels, and the stability of such receptor complexes. Indeed, evidence from different studies has often resulted in distinct views on the extent of quaternary organisation for a single specific receptor. For example, while some studies have suggested that the M_2_ **muscarinic acetylcholine receptor** exists in multiple coexisting and interchanging states, in which monomers predominate at steady state [22], other reports suggest that this receptor exists predominantly, if not exclusively, as dimers [23] or even as tetramers 24, 25, 26, 27. Moreover, while some studies have concluded that protomer–protomer interactions are stable over a broad range of receptor expression levels 23, 28, others have provided data to suggest that increases in receptor density lead to an increase in the size of receptor complexes as a result of transient protein–protein interactions 5, 7, 8, 29, 30. This is consistent with the concept that mass action may play a fundamental role in defining the quaternary structure of these transmembrane proteins 5, 7, 8, 29, 30. Recently, each of these topics has begun to be addressed using SpIDA 1, 5, 6, 7, 8, 9.

The first example of analysis of GPCR quaternary structure using SpIDA was essentially a proof-of-concept study, because it was previously well established that the functional GABA_B_ receptor is a heterodimeric complex formed between coexpressed but distinct GABA_B_R1 and GABA_B_R2 subunits [11]. This study explored the quaternary arrangement of immunocytochemically stained GABA_B_ receptors in sections of rat spinal cord [1]. Performed over a broad range of receptor density, this revealed the presence of identified dimers only when both GABA_B_R1 and GABA_B_R2 subunits were labelled with subunit-specific primary antibodies and detected with the same Alexa-488 fluorophore-conjugated secondary antibody while, by contrast, only monomers were observed when only one of the subunits was labelled [1]. This study established that SpIDA could be used as a tool to characterise the quaternary arrangement of other GPCRs for which less is known about either the extent or the molecular basis of dimerisation.

**Figure 2:**
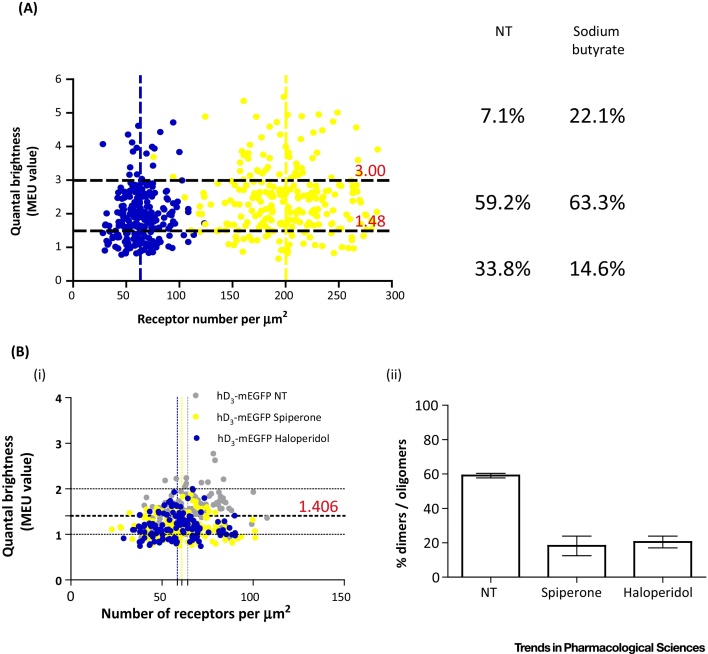
Effect of Expression Level and Ligand Treatment on GPCR Quaternary Structure. (A) The oligomeric state of the secretin receptor–mEGFP expressed in untreated CHO-K1 cells (blue) or cells treated overnight with 5 mM sodium butyrate (yellow). Vertical broken lines represent mean receptors per μm^2^ for control and sodium-butyrate-treated cells. The horizontal dotted lines represent 1.48 and 3.0 MEU respectively (see Ward *et al.*[8] for determination of these values). RoIs characterised by QB MEU >1.48 were considered to contain a prevalence of secretin–mEGFP receptor in larger than monomeric state, while those characterised by QB MEU >3.0 were considered to contain a prevalence of secretin–mEGFP receptor in an oligomeric, rather than dimeric, state. Proportions of outcomes corresponding to monomer, dimer, or oligomer are shown. Data are adapted from Ward *et al.*[8]. (B) The oligomeric state of hD_3_R–mEGFP expressed as QB MEU in HEK293 derived cells untreated (grey) or treated overnight with 10 μM spiperone (yellow) or haloperidol (blue) is shown. Vertical broken lines represent the mean receptor per μm^2^ for untreated and treated cells (i). RoIs characterised by QB MEU >1.406 were considered to contain a prevalence of hD_3_R–mEGFP in a dimeric/oligomeric state (ii). Data are adapted from Marsango *et al.*[7]. Abbreviations: mEGFP, monomeric enhanced GFP; hD_3_R, human dopamine D_3_ receptor; MEU, monomeric equivalent unit; NT, not treated; QB, quantal brightness; RoI, region of interest.

Milligan and collaborators have subsequently made extensive use of SpIDA measurements to define the quaternary arrangement of exemplars of both class A 5, 6, 7 and class B [8] receptors. For all such studies, clearly delineated monomeric and dimeric control constructs are required to define values for QB of the fluorophore of interest, as this is integral to subsequent analysis. In various studies these have included both single molecules and tandemly organised pairs of **monomeric enhanced GFP (mEGFP)** targeted to the plasma membrane by a linked palmitoylation + myristoylation peptide sequence, or the equivalent forms of mEGFP linked to the C-terminal region of the monomeric, single transmembrane domain protein CD86 (Box 1 and Figure 1). To ensure the monomeric state of EGFP within tagged constructs it is routine to introduce an Ala^206^Lys mutation [31]. Initially, the quaternary structure of the **serotonin 5-hydroxytryptamine 2C (5-HT_2C_) receptor** was assessed [5]. Herein, the receptor was modified at the carboxy-terminal tail by in-frame fusion of Ala^206^Lys mEGFP. Following introduction of this construct into the inducible locus of **Flp-In T-REx 293 cells** 5, 32, varying concentrations of the antibiotic doxycycline were used to promote expression of different levels of the receptor. SpIDA measurements were then performed on RoIs selected in laser scanning confocal images of the **basolateral membrane** of such cells. This analysis indicated the receptor to be present in multiple states ranging from monomers to higher-order oligomers and that quaternary complexity of 5-HT_2C_–mEGFP increased markedly with receptor density, such that the receptor was organised mainly as dimers and higher-order oligomers at the highest level of expression assessed [5]. These results were different to conclusions reached using FCS with PCH, which indicated that the same molecular construct as used above was dimeric across a broad range of expression levels [23]. However, the conclusion that class A GPCRs vary in organisational structure with expression level has also been supported for other receptors by others using different approaches 29, 30. Using single-molecule sensitive total internal reflectance fluorescence microscopy (TIRF-M) analysis of fluorescently-labelled **SNAP-tagged** β_1_- and β_2_-adrenoceptors, Calebiro and collaborators [29] concluded that both receptors can be present in mixtures of complexes of different sizes and that the complexity of the quaternary structure increased with receptor density as a result of transient receptor–receptor interactions (lifetime was estimated to be 4 s at 20 °C). In particular, the effect of receptor expression on receptor complexity for the β_1_-adrenoceptor indicated it to be mainly monomeric at low particle density (70:30 monomer:dimer ratio) whereas for the β_2_-adrenoceptor dimers constituted 60% of the total at similar receptor levels, and at higher levels it was predominantly dimeric with a small proportion of tri/tetramers at the highest receptor density assessed [29]. Similar conclusions have been drawn from single molecule analysis of a fluorescently labelled, SNAP-tagged (Figure 1A) version of the D_2_ **dopamine receptor** [30]. Two other studies using SpIDA have also shown increasing quaternary organisation with higher levels of receptor density. The first of these focused on the human D_3_ dopamine receptor [7]. Following constitutive expression in Flp-In T-REx 293 cells of a C-terminally mEGFP-tagged form of the human D_3_ dopamine receptor (hD_3_R-mEGFP), three clones that showed varying levels of receptor expression, as assessed by both western blot analysis of cell lysates and specific binding of the radiolabelled antagonist [^3^H]spiperone, were studied in detail [7]. SpIDA measurements on confocal images taken from the basolateral membrane of cells from each clone showed in each case that, at steady state, a substantial proportion of the RoIs contained receptors predominantly in dimeric/oligomeric states. Interestingly, the dimer to monomer ratio was greatest at the cell surface of the clone with the highest hD_3_R expression level [7]. To exclude that this was not simply an issue of clonal variability cells from a single clone were treated with sodium butyrate; a strategy known to increase the expression level of a number of other GPCRs 33, 34. This increased expression of hD_3_R-mEGFP by 60% and was associated with a substantial increase in the proportion of RoIs in which the receptor was organised predominantly in dimeric/oligomeric states [7]. This approach was also used in the study of oligomerisation of the class B secretin receptor [8]. As stated above, previous studies 13, 14 described the wild-type secretin receptor as existing predominantly if not exclusively in a dimeric state, but that a monomeric state was favoured by the introduction of two single point mutations (Gly^243^Ala and Ile^247^Ala) into TMIV of the receptor 13, 14. To gain further insights into the basis of oligomerisation of the secretin receptor and to assess the proportion of the receptor that might be dimeric, Ward and collaborators performed SpIDA measurements on the basolateral membrane of CHO-K1 cells stably expressing either wild-type or the Gly^243^Ala-Ile^247^Ala mutated secretin receptor; both modified at the carboxyl terminus by addition of mEGFP [8]. This analysis showed that although both types of receptor were expressed at equivalent levels, the quaternary organisation of the wild-type and mutated receptors was different. In particular, although the wild-type secretin receptor was found to be largely dimeric, this was not complete as had been suggested by Harikumar and collaborators [13]; a substantial fraction was monomeric (Figure 2A). By contrast, and indeed as predicted by Harikumar *et al.* [13], the Gly^243^Ala-Ile^247^Ala secretin receptor was recorded to be almost entirely monomeric, with only some 10% being scored as dimeric [8]. Treatment of cells expressing either the wild-type (Figure 2A) or the Gly^243^Ala-Ile^247^Ala secretin receptor with sodium butyrate substantially increased expression level of both forms as assessed by both immunoblotting and fluorescence analysis of confocal images [8]. QB analysis of RoIs from the basolateral membrane of cells showed that sodium butyrate treatment also modified the quaternary structure of the wild-type receptor by increasing its organisational complexity such that >20% of the observations were now scored as oligomeric rather than dimeric (Figure 2A). By contrast, an equivalent effect on the quaternary organisation of the Gly^243^Ala-Ile^247^Ala secretin receptor variant was not observed [8]. These studies also assessed the potential contribution of G protein availability to steady-state receptor dimerisation. Extensive downregulation of the α subunit of G_s_ (the G protein that transduces signal via the secretin receptor to elevation of cAMP levels) did not modulate the extent of dimerisation [8], indicating no key role for the G protein in defining receptor quaternary structure.

## Using SpIDA to Examine Potential Ligand-Induced Alterations of GPCR Quaternary Structure

The most basic and fundamental aspects of GPCR-regulated physiology are driven by the interactions between the receptor and the ligand to which it binds and how this regulates signalling cascades. If the extent of receptor oligomerisation is an important element in the control of responses to either endogenous ligands or synthetic medicines and drugs, then it might be anticipated that this could be altered by ligand binding.

In extending SpIDA-based analysis of the steady-state level of oligomerisation of the human 5-HT_2C_ receptor, it was observed that treatment of cells expressing 5-HT_2C_–mEGFP with several compounds with antagonist/inverse agonist function at this receptor promoted its monomerisation [5]. Importantly, this effect was time dependent and was reversed following washout of the ligands, resulting in restoration of the initial oligomeric organisation [5]. In these studies reversal of the effect of the ligands was assessed 60 min after washout but rate of dissociation of the ligands was not assessed and, as their affinity for the 5-HT_2C_ receptor is high, then ligand ‘off-rate’ could be anticipated to be slow. Future studies should consider using ligands with rapid dissociation rates and attempt to define if quaternary structure is restored rapidly following ligand dissociation. Only three ligands were assessed in these studies and, because two of these were closely related structurally, further studies should also explore a greater diversity of chemotypes. This may be illuminating because in equivalent studies using a broader range of antagonist/inverse agonist ligands at the hD_3_R, as well as identifying ligands that promoted monomerisation of the receptor, several ligands were shown to produce no effect on the monomer/dimer ratio (Figure 2B), [7]. Specifically, both spiperone and haloperidol favoured the monomeric state of the receptor, and this effect was both concentration dependent and, like for the 5-HT_2C_ receptor, reversed upon washout of the drug [7]. One surprising and currently unexplained feature of the effect of spiperone was whilst the induced monomerisation of the receptor was concentration dependent, half-maximal effect required higher concentrations of spiperone than predicted to produce half-maximal receptor occupancy [7]. This clearly requires further analysis but could potentially reflect a much-discussed concept 35, 36, 37 whereby binding of spiperone to a hD_3_R dimer could display significant negative cooperativity, where binding of the first molecule of spiperone would reduce the affinity of a subsequent molecule of spiperone to bind to the second orthosteric pocket. In this situation the observed potency of spiperone suggests that each of the orthosteric binding sites needs to be occupied prior to induced receptor monomerisation. Importantly, the requirement for the receptor to bind (at least one molecule of) spiperone to induce monomerisation has already been demonstrated. Asp^110^Ala hD_3_R is unable to bind spiperone with significant affinity [7]. However, while this mutation did not affect the steady-state extent of dimerisation compared to the wild-type receptor observed at equivalent expression levels [7], treatment of cells expressing this mutant with spiperone did not induce monomerisation of Asp^110^Ala hD_3_R–mEGFP [7].

Unlike spiperone and haloperidol, ligands including eticlopride, the structurally closely related ligand nemonapride, and clozapine had no effect on the oligomeric structure of hD_3_R–mEGFP [7]. To attempt to provide a molecular basis for these observed differences a combination of ligand docking studies and molecular dynamics simulations were used [7]. These suggested that binding of spiperone induces an increase in the distance between reference α-carbon atoms in TMs IV and V, while binding of haloperidol increases the distance between reference α-carbon atoms in TMs I and II but not those in TMs IV and V [7]. Such studies highlight that there must be multiple inactive conformations of a GPCR, and that distinct receptor antagonists/inverse agonists favour different inactive conformations (in a similar vein to the idea that different **agonist** ligands may stabilise distinct active conformations). Although simply correlative at this point, mutagenesis studies designed to define the key regions of TM domains involved in dimerisation of hD_3_R have indicated important contributions of residues within each of TMs I, II, and IV and V [38] (see Box 2 for discussion on the molecular basis of GPCR quaternary structure). That not all antagonist/inverse agonist ligands at the hD_3_R induce measurable alterations in steady-state dimerisation/oligomerisation of this receptor suggests that a more broad-ranging analysis of potential effects of clinically used and trialled antipsychotic drugs would be of interest, as would correlations of their effects on hD_3_R, and potentially D_2_-dopamine receptor, organisation with other clinical parameters.

Box 2 **GPCR Quaternary Structure**

Because GPCRs are characterised by poor protein stability when removed from the lipid-rich environment of a membrane and can assume multiple conformational states, structural studies are challenging. Recently, however, extensive optimisation of crystallisation conditions (i.e., introduction of fusion partners and single point mutations, addition of selective ligands or antibodies during protein purification and crystallisation) has resulted in variants with markedly increased stability more suitable for crystallographic studies and, to date, >130 GPCR structures have been resolved [47]. In some of these, the receptors were revealed as parallel dimers and/or tetramers suggesting the existence of different dimer interfaces for different GPCR homodimers 48, 49, 50, 51, 52, 53, 54, 55, 56. These structures showed rather conserved contact interfaces involving TMI, II and intracellular helix VIII as observed in the β_1_-adrenoreceptor [51], μ- and κ- opioid receptors 48, 50, rhodopsin 54, 55, and opsin [56]. By contrast, less conserved interfaces were observed on the other side of the receptor TM bundle, with TMIV–V interactions observed in β_1_-adrenoreceptor [51], the smoothened receptor [52], and the adenosine A_1_ receptor [53]; TMV–VI interactions observed in the μ-opioid receptor [48]; and mainly TMV–TMV interactions, with contributions of residues also from the first intracellular loop, observed in the chemokine CXCR4 receptor [49].

It is clearly possible that some of these observed interfaces reflect crystal packing artefacts rather than defining physiologically relevant dimer interfaces. As such, distinct approaches are required to support or validate such ideas. As well as a vast range of studies that have explored protein–protein interactions via imaging and resonance energy transfer-based methods [57], predictions from atomic level structural studies have been assessed via both chemical crosslinking and mutagenesis. For example, the two crystallographic interfaces of the β_1_-adrenoreceptor were confirmed to be relevant via receptor crosslinking studies [51]. Furthermore, combinations of molecular modelling, mutagenesis and resonance energy transfer studies have been used to assess the human D_3_ dopamine receptor organisational structure shown to be akin to that of the β_1_-adrenoreceptor dimeric structures [38]. The same approach has also been used to define the interfaces that allow M_3_ muscarinic acetylcholine receptor dimer interactions [58] and resulted in conclusions broadly in agreement with work of others based on both chemical crosslinking experiments 59, 60 and wide ranging, unbiased mutagenesis studies [61]. Finally, contributions of residues within TMI–II–IV–V and helix VII have also been described in studies using cysteine crosslinking that were designed to explore the basis of D_2_ dopamine receptor homodimer formation 28, 62, 63. The existence and potential pharmacological and physiological relevance of GPCR quaternary structure has been reviewed extensively 11, 20, 21.

Alt-text: Box 2

If certain ligands induce monomerisation of hD_3_R and the 5-HT_2C_ receptor then there is no intrinsic reason to assume that other ligands, maybe at different GPCRs, could not increase quaternary complexity. Although systematic consideration is lacking, it is becoming established that the extent of dimerisation differs between class A GPCRs expressed at similar levels and in the same cellular background. This was made explicit by Calebiro *et al.* [29] when comparing β_1_- and β_2_-adrenoceptors. SpIDA of the human muscarinic M_1_ (hM_1_) and hM_3_ receptors revealed both to be present predominantly as monomers at the cell surface, although a significant fraction of dimers was also observed [6]. Sustained treatment of cells expressing M_1_R–mEGFP with the selective M_1_R antagonist/inverse agonist pirenzepine, or the structurally related ligand telenzepine, resulted in both a marked increase in the density of the receptor and in the percentage of RoIs within which the receptor was organised predominantly as dimers/oligomers [6]. Enhanced organisational structure might simply have resulted from the enhanced level of expression and the effect of mass action. However, with M_1_R–mEGFP cloned into the antibiotic inducible locus of Flp-In T-REx 293 cells, levels of M_1_R–mEGFP expression in the basolateral membrane of vehicle-treated cells could be titrated to be akin to those present in pirenzepine-treated samples. SpIDA measurements then showed pirenzepine treatment to have enhanced the proportion of M_1_R dimers independent of receptor upregulation [6]. Although little discussed by later commentators, Ilien *et al.* [39] had earlier used a fluorescence resonance energy transfer (FRET)-based approach to also show an enhanced proportion of M_1_R dimers to be generated rapidly upon binding of pirenzepine. As for the hD_3_R, not all muscarinic antagonists were able to mimic the effect of pirenzepine and telenzepine, as neither atropine nor *N*-methylscopolamine did so [6]. A molecular understanding of these differences remains to be defined. Even closely related receptors can show substantial differences in ligand-induced alterations in quaternary structure. The M_3_ muscarinic receptor is also able to bind both pirenzepine and telenzepine (although with significantly lower affinity than hM_1_R). However at saturating concentrations, neither of these ligands modified the quaternary structure of hM_3_R [6]. Clearly, further work is required to understand the basis of these differences.

To date, virtually all SpIDA-based studies on ligand regulation of GPCR quaternary structure have centred on antagonists/inverse agonists. It might be anticipated that agonist ligands could also alter receptor quaternary structure. Indeed, as proof of concept that this can be monitored and quantified, in a different receptor class, a series of studies have shown that in Flp-In T-REx 293 cells induced to express the epidermal growth factor receptor (EGFR) linked to mEGFP addition of EGF results in rapid transition of the receptor construct from being predominantly monomeric to being largely dimeric 5, 8, 40. However, this did not occur when using a mutant receptor unable to bind EGF effectively [8]. However, only in the case of the secretin receptor has SpIDA been used to date to assess if short-term agonist treatment alters quaternary structure of a GPCR. Here, secretin treatment for a 10-min period did not invalidate the use of SpIDA but no significant agonist effect on receptor organisation was detected [8]. To extend the study of agonist effects on GPCR oligomeric structure, several potential approaches to overcome issues such as internalisation of GPCRs are likely to be used in the near future. An example is the use of cellular systems in which β-arrestins, which play key roles in clathrin-dependent endocytosis, have been eliminated by genome editing [41].

## Concluding Remarks

In this article a brief description of procedures used to perform SpIDA measurements has been given, followed by a detailed description of studies conducted to date in which SpIDA has been used to investigate the oligomeric organisation of GPCRs and its regulation. Such studies have provided new insights into this contentious issue, but many challenges remain (see Outstanding Questions); both in terms of further development of SpIDA, and how information from such studies can be combined with other imaging modalities and/or with approaches, including molecular dynamics simulations of ligand interactions with GPCRs. Given the significant interest in the potential existence of heteromeric GPCR complexes, the development of two-colour SpIDA has recently been described by Godin and co-workers [42], and this is likely to be useful in addressing proportions of GPCR homomers and heteromers present in a sample that coexpresses a compatible pair of GPCRs. Importantly, because SpIDA is based on analysis of simple confocal images, and these can be derived from fixed cells and tissues, it should be possible to perform SpIDA measurements more routinely on tissue sections from mice and other model organisms expressing mEGFP, or other wavelength-shifted, fluorophore-tagged GPCRs of interest. In this way GPCR oligomerisation state could be explored in physiologically relevant settings and contexts, in animal models of disease or following the administration of therapeutic drugs.

## Disclaimer Statement

The authors declare no conflicts of interests.

Outstanding Questions **Does Agonist Binding Modify Receptor Quaternary Structure?**

In general, agonists promote receptor clustering and internalisation, making confocal images difficult to analyse by SpIDA. Consequently, either the time of treatment with ligand must be limited or alternative approaches such as expression in cell lines genome edited to prevent agonist-induced internalisation and/or clustering of receptors to clathrin-coated pits could be adopted.

**What Role Does G Protein-Coupling Play in Defining GPCR Quaternary Structure?**

Few studies have focused on the mechanisms of interaction between the protomers of a GPCR oligomeric complex and G proteins, and it is unclear whether GPCR quaternary structure is affected or defined by G protein coupling. SpIDA could be performed on cells previously treated with molecules that inhibit G-protein coupling or are genome edited to lack expression of specific or multiple G proteins.

**Can SpIDA Be Used to Study the Kinetics of Formation and Stability of GPCR Oligomers?**

SpIDA could be used, in combination with other methods such as TIRF-M, to obtain information about protomer–protomer association and dissociation constants.

**Can SpIDA Be Used to Detect and Quantify GPCR Hetero-oligomers?**

To date, SpIDA has only been used to observe and quantify GPCR homo-oligomers. However, two-colour SpIDA [42], using pairs of fluorescent proteins with similar excitation but different emission wavelengths, is being developed and is likely to allow concurrent detection and quantification of homo- and hetero-oligomeric interactions.

**Can SpIDA Be Used to Monitor Receptor Quaternary Organisation in Native Tissues?**

Although already reported using fluorescent antibody labelling, SpIDA can potentially be used with tissue sections from transgenic animal models expressing receptors suitably tagged with a monomeric fluorescent protein. Such studies will allow analysis of receptor oligomerisation and its response to ligand binding in an *ex vivo* physiological context. Potential links to ‘light sheet’ and other deep tissue microscopy methods may also allow such studies to be extended into intact tissue from such animal models.

## Glossary

**Agonist**: chemical species that binds to a receptor and activates it, eliciting a biological response.
**Antagonist**: chemical species that binds to a receptor preventing activation by an agonist.
**Basolateral membrane**: applied here to adherent cells in culture and refers to the part of the membrane in contact with the growing surface, in this case a glass coverslip.
**Laser scanning confocal microscope images**: technique for recording optical sections through a fluorescently labelled sample using laser illumination to excite the sample and a pinhole to exclude out of focus light.
**Dopamine receptors**: class A GPCRs whose primary endogenous ligand is the neurotransmitter dopamine. They are involved in many neurological processes, including motivation, pleasure, memory, learning, and fine motor control.
**Flp-In T-REx 293 cells**: inducible expression system in which a cDNA construct of interest is integrated into a defined locus in the genome, under the control of the tet repressor. Addition of tetracycline (or the more stable doxycycline) binds the tet repressor protein allowing protein expression from the integrated construct.
**G protein-coupled receptor (GPCR)**: integral membrane protein able to transmit a signal across the cell membrane in response to an external stimulus, to activate (usually) heterotrimeric guanine nucleotide binding proteins (G-proteins). Characterised by a conserved structure comprising seven helical membrane-spanning domains, an extracellular N terminus, three external loops, three internal loops, and an internal C-terminal domain. Subdivided into classes based upon structural relatedness.
**Inverse agonist**: chemical species that binds to a receptor and reduces its ligand independent or constitutive activity.
**Monomeric enhanced GFP (mEGFP)**: GFP isolated from the jellyfish Aequorea victoria, mutated to enhance stability and brightness and ensure monomeric character (A206K). It may be fused to proteins of interest as a label allowing visualisation of the cellular location of the protein of interest.
**Muscarinic acetylcholine receptors**: a small family of five GPCRs whose endogenous ligand is acetylcholine.
**Quantal brightness (QB)**: average value assigned by the SpIDA software to the relative brightness of an RoI within a cell expressing a fluorescently labelled protein.
**Secretin receptor**: a GPCR for which the endogenous ligand is the 27-amino-acid secretin peptide.
**Serotonin 5-hydroxytryptamine 2C (5-HT_2C_) receptor**: also known as 5-hydroxytryptamine receptors, this family of 16 GPCRs (and also a single ligand-gated Na^+^/K^+^ cation channel) is found in the central and peripheral nervous systems. They mediate both excitatory and inhibitory neurotransmission.
**SNAP-Tag**: self-labelling protein tag, based upon the DNA repair enzyme *O*-6-methylguanine-DNA methyltransferase. Covalently interacts with benzylguanine-linked species that allow incorporation of molecules of interest, including fluorescent dyes.
**Spatial intensity distribution analysis (SpIDA)**: an approach and image analysis tool for determining the oligomerisation state of a fluorescently labelled protein.
**Super-Poissonian distribution function**: a probability distribution having a larger variance than a Poisson distribution but with the same mean value.
